# In Vitro Study of the Biological Potential of Wastewater Obtained after the Distillation of Four Bulgarian Oil-Bearing Roses

**DOI:** 10.3390/plants11081073

**Published:** 2022-04-14

**Authors:** Yana Ilieva, Lyudmila Dimitrova, Almira Georgieva, Neli Vilhelmova-Ilieva, Maya Margaritova Zaharieva, Zlatina Kokanova-Nedialkova, Ana Dobreva, Paraskev Nedialkov, Vesselin Kussovski, Alexander D. Kroumov, Hristo Najdenski, Milka Mileva

**Affiliations:** 1 Department of Infectious Microbiology, The Stephan Angeloff Institute of Microbiology, Bulgarian Academy of Sciences, 26 Acad. G. Bonchev Str., 1113 Sofia, Bulgaria; illievayana@gmail.com (Y.I.); lus22@abv.bg (L.D.); zaharieva26@yahoo.com (M.M.Z.); vkussovski@gmail.com (V.K.); hnajdenski@gmail.com (H.N.); 2 Department of Virology, The Stephan Angeloff Institute of Microbiology, Bulgarian Academy of Sciences, 26 Acad. G. Bonchev Str., 1113 Sofia, Bulgaria; almirageorgieva@gmail.com (A.G.); nelivili@gmail.com (N.V.-I.); 3 Institute of Neurobiology, Bulgarian Academy of Sciences, 1113 Sofia, Bulgaria; 4 Pharmacognosy Department, Faculty of Pharmacy, Medical University of Sofia, 2 Dunav str., 1000 Sofia, Bulgaria; zlatina.kokanova@pharmfac.mu-sofia.bg (Z.K.-N.); pnedialkov@pharmfac.mu-sofia.bg (P.N.); 5 Department of Aromatic and Medicinal Plants, Institute for Roses and Aromatic Plants, Agricultural Academy, 49 Osvobojdenie Blvd, 6100 Kazanlak, Bulgaria; anadobreva@abv.bg; 6 Department of Biotechnology, The Stephan Angeloff Institute of Microbiology, Bulgarian Academy of Sciences, 26 Acad. G. Bonchev Str., 1113 Sofia, Bulgaria; adkrumov@gmail.com

**Keywords:** rose wastewaters, antiradical activity, antiherpesvirus activity, cytotoxicity, antibacterial properties

## Abstract

The wastewater after rose oil distillation is usually discharged into the drainage systems and it represents a serious environmental problem. While being rich in polyphenols, which have beneficial biological activity and application in the pharmaceutical industry, limited research has been carried out about the biological activity of the specific wastewaters per se. Wastewaters after distillation of the four Bulgarian oil-bearing roses *Rosa damascena* Mill., *R. alba* L., *R. centifolia* L., and *R. gallica* L. exerted significant antioxidant activity and good antiherpes simplex virus type-1 (HSV-1) activity while maintaining a good toxicological safety profile (low cytotoxic effect) towards normal cell lines. More precisely, the non-tumorigenic cells were a human (HEK-293 embryonic kidney cells) and a mouse cell line (CCL-1 fibroblasts, which are recommended as a standard for cytotoxicity evaluation in Annex C of ISO 10993-5). The concentrations that achieved antioxidant and radical scavenging effects (0.04–0.92% *v*/*v*) were much lower than most of the maximum tolerated concentrations for the tissue culture cells (0.2–3.4% *v/v*). The wastewaters had a weak antiproliferative effect against *Staphylococcus aureus*. None of the wastewaters had activity against Gram-negative bacteria or a bactericidal or antifungal effect. We can conclude that these four species, which are the most preferred species worldwide for producing high-quality rose oil, have the potential to be developed as promising antioxidant and antiherpesvirus nutraceuticals.

## 1. Introduction

The representatives of the Rosaceae family *Rosa damascena* Mill, *Rosa gallica* L., *Rosa centifolia* L., *Rosa alba* L., and *Rosa rugosa* Thunb. are the most preferred species worldwide for producing high-quality rose oil [1,2]. The world leaders in rose production are countries such as Turkey, China, countries of the former Soviet Union, Egypt, Morocco, Saudi Arabia, and Bulgaria [3,4,5]. In Bulgaria, the cultivation and processing of roses is not only very important for the country’s agricultural economy but also a tradition and the livelihood of a large part of the population. *R. damascena*, *R. alba*, *R. gallica*, and *R. centifolia* are the four rose species that are grown predominantly and are most widespread [5,6].

There is a lot of historical evidence that since ancient times, roses have been used not only as ornamental plants with beautiful colors and aromas, and for religious ceremonies and weddings, but also for preventive and therapeutic purposes. Water, ethanol, and methanol extracts have anti-HIV [7], antitussive [8], bronchodilatory [9], antioxidant, and antimicrobial [10] effects. Ethanolic extract from the flower parts of *R. centifolia* has a beneficial effect on the skin in bites and stings, as well as in burns, on the respiratory and digestive systems, in eye diseases, and cancer [11]. In Turkish folk medicine, *R. gallica* roots were used internally against hemorrhoids [12]. *R. gallica* petal 70% ethanol extract showed anti-skin inflammatory effects through the suppression of UV-induced MAPK (mitogen-activated protein kinase) activation [13], and 50% ethanol extract exhibited an anti-aging effect [14]. It was proved in vivo that 70% ethanol extract had an anti-stress effect in mice [15].

The most popular method for producing rose oil is a classical method of water–steam distillation of the flowers, and the water fraction is considered a waste material of the technological process. Farmers process roses into essential oil or rose water, but they are alarmed that the wastes are in large quantities. According to Kovacheva et al. (2010) [16], during an industrial distillation, 500 to 1000 kg of rose petals are used. The distillation of 1 kg of raw flower material leads to 4 L of liquid waste. The wastewater after rose oil distillation is rich in polyphenols, which are difficult to decompose. Most often they are discarded into the drainage system and the rivers and, for that reason, they represent a serious environmental problem as bio pollutants [17]. On the other hand, there are much data on the beneficial biological activity of polyphenols and their application in the pharmaceutical and cosmetic industries.

In the scientific literature we found that very limited data on the chemical characterization of the wastewater after rose production are established. It is reported that during the distillation process, the non-volatile phenolic compounds remain in the wastewater, such as tannins [18], flavonoids [19], anthocyanins [20], triterpenoids [21], and other polyphenols. The polyphenolic compounds are known to have a wide spectrum of biochemical and pharmacological effects, such as the prevention of cardiovascular diseases, cancers, osteoporosis, neurodegenerative diseases, and diabetes mellitus [17,22]. Kaempferol and quercetin (bioflavonoids) were previously found in the flower extracts of *R. rugosa* [23] and kaempferol, quercetin glucosides, galactosides, arabinosides, and rhamnosides in *R. damascena* petals [24]. Quercetin is a natural catechol-bearing compound that suppresses lipoxygenase activities [25,26]. Kaempferol has not only antioxidative and antitumor properties but also anti-inflammatory effects by suppressing NF-κB signals [27]. Gallic acid derivatives and polysaccharides from the aqueous extract of R. rugosa flowers have antioxidant properties [28]. Thus, the rose WWs (wastewaters) can be used as a good, natural, inexpensive source of biologically active compounds and high-value products after more phytochemical, in vitro, and in vivo studies.

In our previous research, the efforts were focused on the chromatographic profile, redox-modulating capacity, and antineoplastic activity of the wastewaters of *R. damascena*, *R. gallica*, *R. centifolia*, and *R. alba* [29]. We applied UHPLC-HRMS (ultra-high-performance liquid chromatography-high-resolution mass spectrometry) for chromatographic analysis of rose wastewaters, studied their metal-chelating and Fe (III)-reducing ability, and performed MTT (methylthiazolyl tetrazolium) assay for the evaluation of cytotoxic potential against three tumorigenic (HEPG2—hepatocellular adenocarcinoma, A-375—malignant melanoma, A-431—non-melanoma epidermoid squamous skin carcinoma) and one non-cancer human cell line (HaCaT—immortalized keratinocytes).

**Figure 1:**
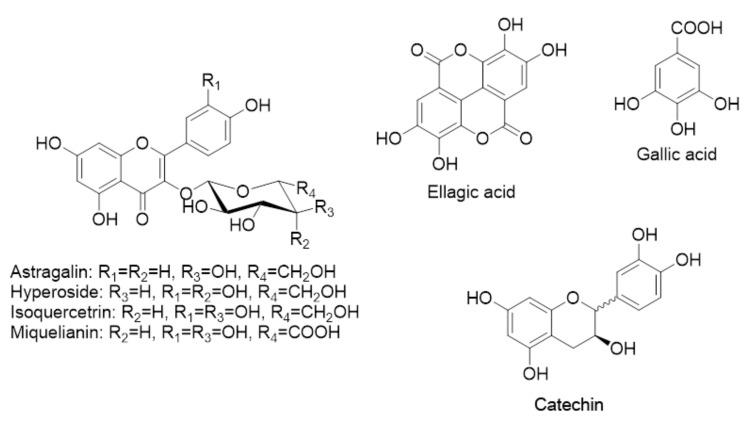
Structures of the main components found in rose wastewaters.

Over 60 compounds in wastewater produced after steam distillation of the flowers of the four rose species were found by our team (in accordance with Georgieva et al., 2021 [29]). Flavonoids, derivatives of ellagic and gallic acids, catechin, epicatechin, and their derivatives were identified. The detected flavonoids showed great structural diversity, with the most common being mono- and glycosides of kaempferol and quercetin and, less commonly, their acylated derivatives. The main flavonoids in the wastewaters of these four roses were astragalin (kaempferol-3-O-glucoside), hyperoside, isoquercitrin, and miquelianin. Ellagic and gallic acid were also found in large quantities. The former was the major component in *R. gallica*, *R. alba*, and *R. centifolia* wastewaters. Derivatives of ellagic and gallic acids were in smaller quantities compared to their free forms. Catechin was detected in larger quantities only in wastewater from *R. gallica*. The structures of the main components found in rose wastewaters are given in Figure 1

As can be seen in Figure 1, the wastewaters from the four roses are rich in components with valuable bioactivities. Hence, we studied a panel of bioactivities and found that the WWs from four different *Rosa* spp. induce the executor caspase 3/7 in tumorigenic cell lines but not in “normal” cells. We established a promising redox-modulating capacity of the four wastewaters as excellent cleaners of heavy metal ions. The obtained interesting results led us to a subsequent more detailed study of the radical scavenging activity, and antimicrobial and antiviral effects of the wastewaters.

The present work was formed as a continuation of our previous research with the purpose—to establish the antiradical, antimicrobial, and antiviral potentials of wastewaters from the distillation of the essential oil of four Bulgarian oil-bearing roses: *R. damascena*, *R. alba*, *R. centifolia*, and *R. gallica,* as well as their cytotoxic effect on normal non-tumorigenic cell lines.

## 2. Results

### 2.1. Determination of Total Polyphenols in Wastewaters (WWs)

**Figure 2:**
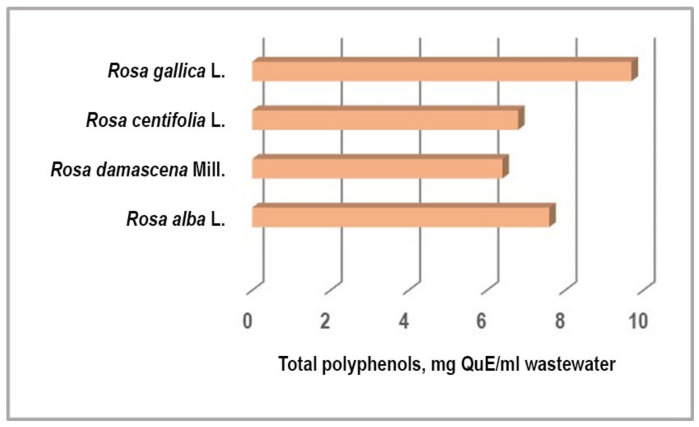
The content of total polyphenols in rose wastewaters, expressed as quercetin equivalent (mg QuE/ ml WWs).

The total polyphenols content expressed as quercetin varied from 6.2 to 9.4 mg/mL in rose wastewater (Figure 2). *R. gallica* and *R. alba* wastewaters demonstrated the highest quantity of polyphenols (9.4 mg/mL and 7.6 mg/mL, respectively).

### 2.2. Antioxidant and Radical Scavenging Properties of WWs

**Figure 3:**
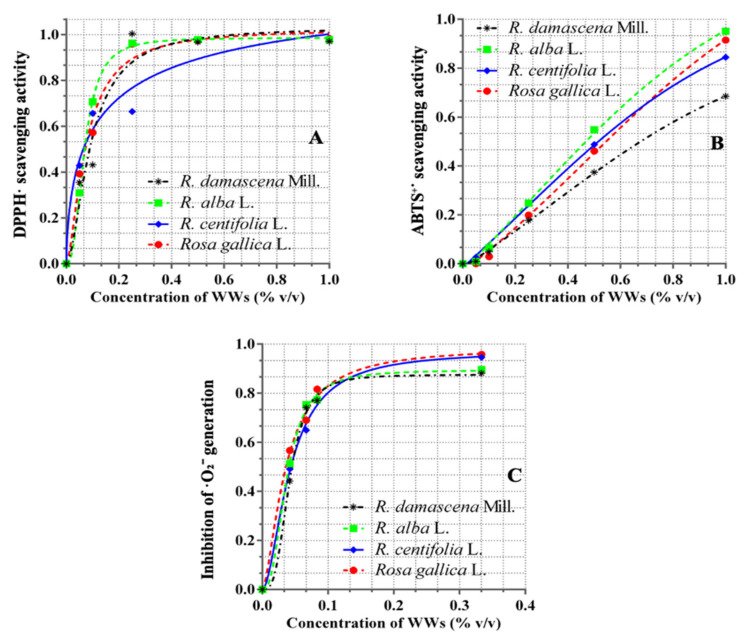
Antiradical activities of rose WWs. (**A**) DPPH scavenging activities of WWs. As a maximal effect (1 equal to 100%), the activity of ascorbic acid at a concentration of 1.136 mM (0.02 mg/mL) has been accepted. The other data were calculated as a part of this activity and expressed as mM ascorbic acid equivalents (mM AaE). (**B**) Data are expressed as mM Trolox equivalents (mM TE). The maximal acceptance of ABTS^+^ (96.9%) was observed at a concentration of Trolox 0.3 mM. The other data were calculated as a part of this activity and expressed as mM TE. (**C**) Inhibition of ^•^O_2_^−^ generation in a system of NBT. As a maximal effect (1 equal to 100%), the activity of ascorbic acid (95.8%) at a concentration of 0.07 mM has been accepted. The other data were calculated as a part of this activity (in mM AaE).

As we can see in Figure 3a, at concentrations of WWs in the range from 0.05% to 0.2%, the highest DPPH (2,2-diphenyil l-picrylhydrazyl) scavenging effect was achieved by WWRa (wastewater from *R. alba*)(0.95 mM AaE) at 0.2%, followed by equal values of WWRg (wastewater from *R. gallica*) and WWRd (wastewater from *R. damascena*), (0.85 AaE) at the same concentrations, and WWRc (wastewater from *R. centifolia*)showed the lowest effect (0.75 mM AaE). At a concentration of 0.25% *v/v* of WWRa, the effect was maximal (0.98 AaE), and remained the same when raising the concentration. Maximal and equal effects (0.97 mM AaE) and their saturation were observed at the range higher than 0.5% *v*/*v* concentration of WWRd and WWRg.

As shown in Figure 3a–c, the antiradical activity of all four effluents shows a strict dose-response relationship. With respect to the superoxide radical, the maximum scavenger effects are reached in concentrations of 0.1% BBC, for DPPH up to 0.2% *v*/*v* of WWs, and the abstinence activity of ABTS^+^ (2,2′-azino-bis (3-ethylbenzthiazoline-6-sulphonic acid) increases gradually in the concentration range of 0.0–1.0% *v*/*v*.

**Figure 4:**
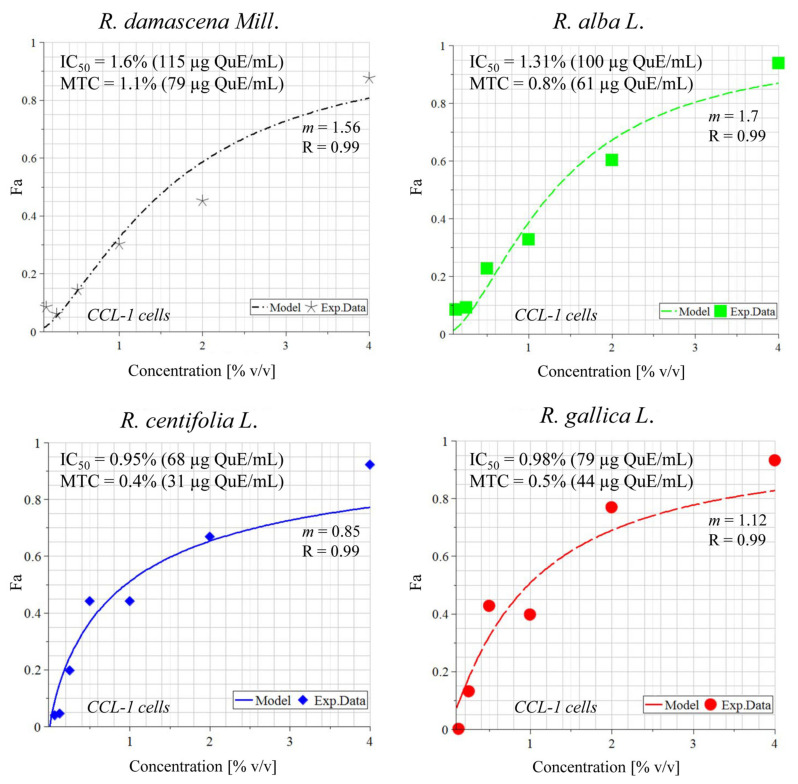
In vitro cytotoxicity of wastewaters obtained from different rose species in CCL-1 cells after 72 h of incubation. Legend: Fa—effect; IC_50_—median inhibitory concentration; MTC—maximum tolerated concentration; m—hillslope; R—coefficient of correlation; QuE—quercetin equivalents; Model—fitting curve; Exp. Data—experimental points.

As can be seen in Table 1, the median effective concentration of the antiradical effects of wastewaters obtained after the distillation of rose oil with respect to all three radicals is shown by WW from *R. centifolia*. As shown in Figure 4, the antiradical activity of all four effluents showed a strict dose-response relationship. With respect to the superoxide radical, the maximum scavenger effects are reached in concentrations of 0.1% BBC, for DPPH up to 0.2% *v*/*v* of WWs, and the abstinence activity of ABTS^+^ increases gradually in the concentration range of 0.0–1.0% *v*/*v*.

### 2.3. Antimicrobial Activities

MIC (minimal inhibitory concentration) and MBC (minimal bactericidal concentration) are calculated from the content of total polyphenols in wastewater (mg/mL). If MIC is detected, then the DEHA (dehydrogenase) activity is practically equal to the untreated control. From all test microorganisms, *S. aureus*, as a representative of Gram-positive bacteria, was typically most susceptible (Table 2). No effect was observed against *P. aeruginosa*, *E. coli*, and *C. albicans*. Wastewater (WW) from the distillation process of *R. alba* petals inhibited the proliferation of *S. aureus* with about 68%, WW from *R. damascena* with about 71%, WW from *R. centifolia* with 55%, and WW from *R. gallica* with 64% at MICs. Interestingly, the most active wastewater was from the distillation of *R. centifolia* oil (MIC=1.9 mg/mL), although the percent of metabolic activity of bacterial cells was high (DEHA = 45.28% ± 0.05). None of the wastewaters had a bactericidal or antifungal effect.

### 2.4. In Vitro Cytotoxicity Evaluation (MTT Assay)

For the toxicological assessment in our work, non-tumorigenic cell lines were used. One human cell line (HEK-293, embryonic kidney cells) and one mouse cell line (CCL-1 fibroblasts, which are recommended as a standard for cytotoxicity evaluation in Annex C of ISO 10993-5:2009) were chosen [30]. Wastewaters from the four rose species had low cytotoxicity towards the two normal cell lines. As usual, the half maximal inhibitory concentration (IC_50_) and the maximum tolerated concentrations (MTC), with some exceptions, were inversely proportional to time–these values decreased with increasing time of incubation.

**Figure 5:**
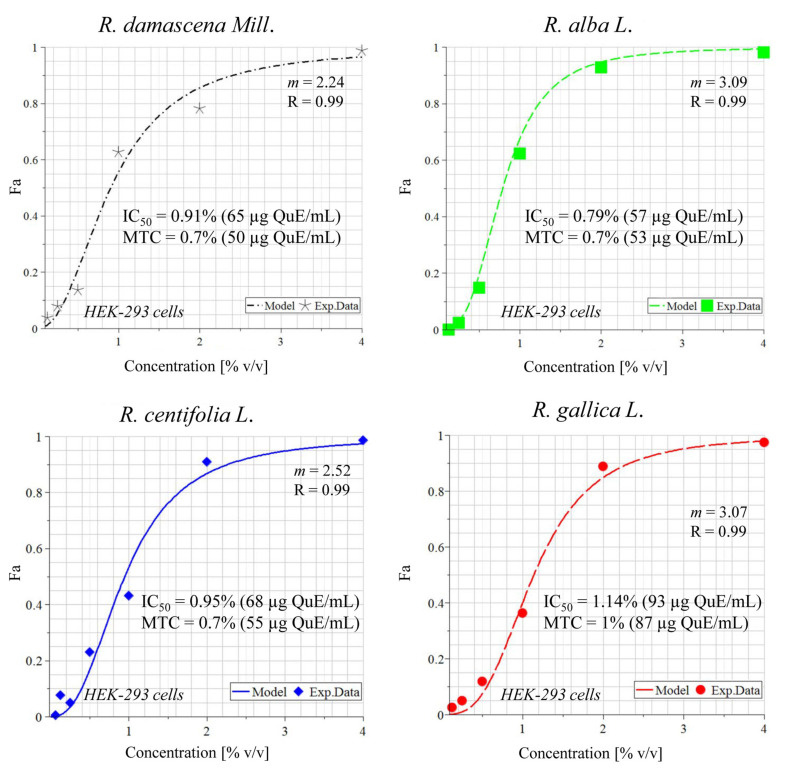
In vitro cytotoxicity of wastewaters obtained from different rose species in HEK-203 cells after 72 h of incubation. Legend: IC_50_—median inhibitory concentration; MTC—maximum tolerated concentration; m—hillslope; R—coefficient of correlation; QuE—quercetin equivalents.

MTCs, or in simple terms, the concentrations that killed only 30% of the cells, ranged from 1.7–3.4% (129–265 µg QuE/mL) for 24 h, 0.2–1.6% (15–125 µg QuE/mL) for 48 h, and 0.4–1.1% (31–79 µg QuE/mL) for 72 h incubation. The IC_50_ values, or the concentrations that killed 50% of the cells, varied between 2.2–11.1% (168–867 µg QuE/mL) for 24 h, 0.8–1.9% (63–146 µg QuE/mL) for 48 h, and 0.8–1.6% (57–146 µg QuE/mL) for 72 h (Table 3, Figure 4 and Figure 5). It is notable that CCL-1 cells were more resistant than HEK-293 at 24 h (with the exceptionally high IC_50_ of 867 µg/mL polyphenols) but were a little more sensitive at 48 h. For the 72 h incubation, no correlation was observed, as well as generally between the species wastewater and activity.

### 2.5. Antiherpes Viral Activities

Table 4 shows that the four tested products had similar cytotoxicity, with the lowest toxicity showing in the wastewater from *R. alba* and the highest in the wastewater from *R. centifolia* (which was almost two times more toxic than that of *R. alba*). At the maximum tolerated concentration (MTC), all four products showed the same value of 0.1%.

The antiherpes viral activity of wastewater against the replication of two strains of herpes simplex virus type 1, Victoria and R-100, in the monolayer cell culture MDBK (Madin-Darby bovine kidney) was studied. All four wastewaters showed weak antiherpes activity against both strains. Inhibition in the Victoria strain was slightly more pronounced. The most significant was the antiherpes effect of *R. gallica* (SI = 12.5) and *R. centifolia* (SI = 9.3) against the Victoria strain. Their effect was close in importance to the replication of the resistant strain with *SI* values for *R. centifolia* (SI = 8.7) and *R. gallica* (SI = 8.3). The weakest antiherpes activity against both tested strains was demonstrated by *R. damascena* wastewater (Table 4).

## 3. Discussion

In this work we supplement our research on the biological activities of the so-called “old roses”—*R. damascena*, *R. alba*, *R. centifolia*, and *R. gallica*. Nazarenko and coworkers (1983) [31] suggest that the genus *Rosa*, to which these oil-bearing species belongs, originates from the ancient evergreen lianas of the Sundarbans in India. This area is called “the pharmacy of the world” as more than a quarter of the drugs known today in medicine are based on plants from these forests [32]. Despite their common ancestors, the four roses in this investigation differ in color, the morphology of flowers and germplasm,, the content of their essential oils, hydrosols, waters, and water-alcoholic extracts, as well as their biological effects [33].

As a rule, healthy cells and tissues are characterized by low levels of reactive oxygen species (ROS) in their normal metabolic functions. Overproduction of ROS above a certain “critical” level provokes disabilities in cells and tissues. To maintain optimal cellular homeostasis in living organisms, the regulation of cellular redox status is extremely important [34,35].

The WWs are rich in polyphenols, which are among the most common secondary metabolites in the plant kingdom, involved in plants’ pigmentation and especially their antioxidant defense system against UV light and different pro-oxidants pollutants [36,37]. Chemically, polyphenols are largely planar molecules and can be divided into several classes, e.g., phenolic acids, flavonoids, isoflavonoids, proanthocyanidins, anthocyanins, stilbenes, and lignans [38], that are known as good natural antioxidants. Despite the benefits of polyphenols, in nature they act as bio pollutants. They regulate processes in the soil by the inhibition of nitrification [39], decomposition, and nutrient recycling [40,41].

In principle, the antioxidant effects of complex plant extracts, such as rose WWs, can include various mechanisms of antioxidant defense—as free radical scavenging, the termination of oxidative chain reactions, reducing capacity, and binding of prooxidant metal ions [42].

According to our previous investigations, WWs might perform an essential detoxification function against ions of copper and iron and, in this way, protect the cellular structures against the damaging effects of reactive oxygen species [29]. In this study, three different assays were employed to evaluate the antiradical properties of the WWs as well as to elucidate their mode of action.

The obtained results indicated that the WWs have very good radical scavenging activities (Table 1). All the samples of WWs showed dose-dependent increases in activity in all tested systems: DPPH radical scavenging, superoxide catching, and ABST^+^ neutralizing. Based on the literature, the mechanisms of reduction of the free radicals are correlated with the presence of hydroxy groups at the molecule. In this context, WWs can be considered a multicomponent antioxidant system that provides different parts of the antioxidant protection strategy and are generally more effective than the pure compounds due to the resulting interactions between different antioxidant components.

Essential oils of roses and some extracts and extract fractions (e.g., ethanol) of *R. damascena* and its variety Taif rose have low to significant cytotoxic effects towards normal cell lines, as described in our recent review [33]. However, there are few studies on the effects of rose wastewaters, water byproducts, water-soluble compounds, or even aqueous extracts from all *Rosa* spp., and even fewer among them study their cytotoxic activity. Most of them are dedicated to *R. damascena* [29,33].

The low cytotoxic effect towards normal cell lines, established in this study, is beneficial for the toxicological safety profile of the wastewaters from the four species. However, the MTC and IC_50_ values for the normal eukaryotic cell lines were smaller than the MIC values for the bacteria (*S. aureus*), meaning that the wastewaters are not suitable for all routes of administration if an antibacterial effect is aimed for. As to our knowledge, there are no data about the antibacterial effect of WWR in the scientific literature. Evaluation of the antimicrobial activity of the wastewaters in our study reveals only a moderate activity on the Gram-positive species *S. aureus*. No activity was observed on the Gram-negative strains tested or the pathogenic fungi *C. albicans*. Therefore, further investigations are needed to explore the full potential of wastewaters for application in products with antibacterial properties, but, in this case, the research should be focused on the possibilities for local administration after careful in vivo tests for skin tolerance due to the higher concentrations that are needed to achieve the desired antibacterial effect. However, in terms of antioxidant activity, the results are favorable—the concentrations that achieve antioxidant and radical scavenging effects are lower than most of the maximum tolerated concentrations. More precisely, only the ABST^+^ EC_50_ (half maximal effective concentration) values (0.69–0.92%) are higher than some MTCs (0.2–0.7%) and the latter values are distributed predominantly among CCL-1 cells and for the longer incubations, i.e., 48 and 72 h (only 72 h for HEK-293). The other of the EC_50_ values (0.04–0.14%) are much lower than all MTCs for the 24th hour for both cell lines (1.7-3.4) and lower than most of the MTCs on HEK-293. It is probable that the good radical scavenging activities, especially of WWRa, regarding the DPPH and ABTS radicals, which have little relevance in live/organism systems, also manifest in a relative harmlessness for cell systems. The inhibition of superoxide generation could be involved in the protection of cell membranes from oxidative damage in vivo. Therefore, the wastewaters studied in this work have shown potent antioxidant activity and are a potential source for the development of promising antioxidant nutraceuticals for both oral and cutaneous administration.

HSV is responsible for a broad range of human infectious diseases. Therapy and the prevention of HSV infectivity are the main goal for many researchers worldwide since herpes virus infections cannot be managed by vaccination. The main classes of antiviral drugs can be classified as virucidal, immunomodulatory, and antiviral chemotherapeutic agents. There are several selective and effective drugs such as acyclovir, penciclovir, famciclovir, cidofovir, valacyclovir, trifluridine, and vidarabine, which act as inhibitors of viral DNA synthesis [43,44]. Unfortunately, the intensive use of these drugs in therapy has led to undesirable effects such as drug-resistant strains [45]. Acute and recurrent HSV infections remain an important problem. For that reason, the search for new antiviral agents and new approaches for the treatment of HSV infection has gained much interest in recent years. In addition, it is an urgent issue to find new therapeutic agents with different mechanisms of action than those of nucleoside analogs. Consequently, natural products are a real treasure, rich in promising sources of new antiviral substances. Therefore, we investigated the ability of the rose wastewaters to inhibit the development of the herpes virus and compared it with that of the most widely used chemotherapeutic agent acyclovir.

Our previous research indicated that the selective index of ACV (acyclovir) in the same experimental cell system was SI= 881.8 for the Victoria strain and SI = 18.6 for R-110 [46,47]. The selective index (SI) is an important criterion for evaluating antiviral activity. It is the ratio between the 50% cytotoxic concentration and 50% effective concentration. If the SI value is more than 4, the substances are considered to have an antiviral effect [48].

Under the same experimental conditions, all four wastewaters indicate the presence of antiherpes activity (Table 4). What they have in common is that the four wastewaters can serve as important raw materials for producing cytotoxically and genotoxically safe commercial products. The inhibition of the Victoria strain is a little better expressed compared to R-100 for the four wastewaters. The most significant is the antiherpes effect of *R. gallica* (SI = 12.5) and *R. centifolia* (SI = 9.3) against the Victoria strain. Their effect is close to that of *R. centifolia* (SI = 8.7) and *R. gallica* (SI = 8.3) regarding the replication of the resistant strain. The weakest antiherpes activity against both tested strains was demonstrated by *R. damascena* wastewater (Table 4). The results on the antiherpesvirus activities of the wastewaters could be explained by the content of polyphenols (Figure 3). The better antiherpes activities of the waters obtained after the distillation of *R. centifolia* and *R. gallica* correlate with their higher polyphenols content.

Previous results showed that the studied rose WWs are rich in quercetin-like flavonoids (Figure 1). The latter are a group of low molecular weight phenyl benzopyrones that have various pharmacological properties including antioxidant, anticancer, bactericidal, and anti-inflammatory effects. Flavanols, which are derivatives of quercetin, show various cytopathic effects on herpes virus-infected Vero cells, as well as selective indices in a wide range [48]. Wastewaters from rose oil production are phytocomplexes, rich in water-soluble components, meaning that their biological activities are due to the overall action (complementary or antagonistic) of all their ingredients. What they have in common is that the four wastewaters can serve as important raw materials for producing cytotoxically and genotoxically safe commercial products [49]. The obtained results allow us to view *R. gallica* and *R. centifolia* as potential antiherpes agents. It is notable that in this study, we used native water without further processing.

## 4. Materials and Methods

### 4.1. General

Hyperoside (≥ 97%, HPLC), pyrogallol (≥ 98%, HPLC), Folin–Ciocalteu’s phenol reagent, AlCl_3_, Na_2_CO_3_ were purchased from Sigma-Aldrich (Taufkirchen, Germany). The measurements for the determination of tannins and total flavonoids were carried out using a Biochrom Libra S70 UV-VIS spectrophotometer (Cambridge, UK).

### 4.2. Preparation, Polyphenol, and Antioxidant analysis of Wastewater from the Industrial Cycle of Water–Steam Distillation of R. damascena Mill., R. alba L., R. gallica L., and R. centifolia L. Oils

#### 4.2.1. Preparation

**Figure 6:**
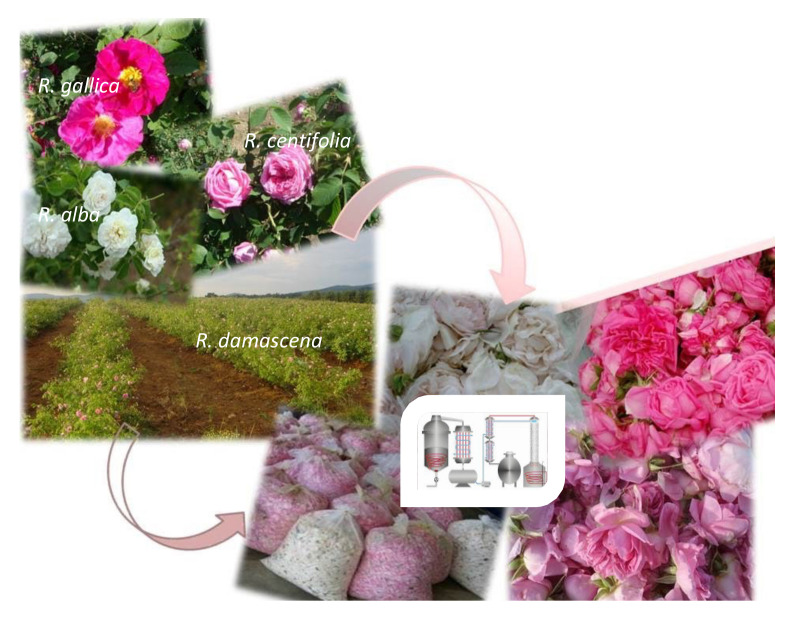
Oil-bearing roses in Bulgarian plantations: production and storage.

**Scheme 1:**
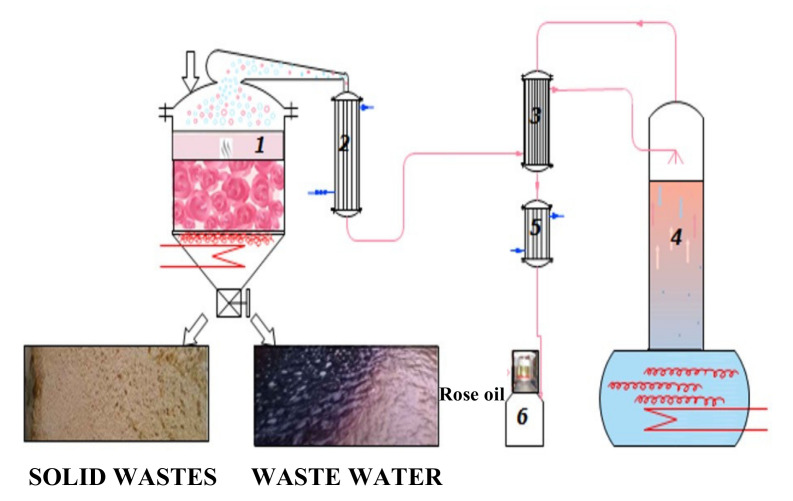
Wastewater production. 1—Distiller; 2,5—Condenser; 3—Heat exchanger; 4—Cohobator; 6—Florentine flask (separator).

The production of rose oil begins with the cultivation of plantations, collection, and storage of rose petals (Figure 6). The next step involves a batch water–steam distillation. The liquid samples were collected during the 2021 rose oil distillation campaign immediately after discharging the distillers at the end of each cycle, as illustrated in Scheme 1.

The semi-industrial installation of the Institute for Roses was used. The process parameters were as follows: raw material 8–10 kg; hydromodul 1:4; flow rate 16–20 mL/min; duration 150 min. The wastewaters were sealed and saved at a cool place until the next stage of the investigation.

#### 4.2.2. Total Polyphenols Determination

This was completed using the method of Singleton et al., 1999, [50] and the content was expressed as mg quercetin equivalent (QuE) per ml WWs.

#### 4.2.3. Antioxidant and Radical Scavenging Properties of Wastewater

##### DPPH Assay

DPPH assay was performed by the method of Brand-Williams et al. (1995) [51]. Dissolved in methanol, DPPH is a stable radical because of the delocalization of the electron on the whole molecule, which prevents the dimerization characteristic for most other radicals. Delocalization of the electron results in saturated violet color was performed, with absorption at 517 nm. In the presence of a substance that can donate a hydrogen atom, DPPH is converted to 2,2-diphenyil l-picrylhydrazine, which is accompanied by a fading of the dark violet solution. The electron donor properties of studied rose products were determined by the transition of the violet methanol solution of DPPH to yellow. The wastewater solution was diluted in methanol as follows: 10 μL, 20 μL, 50 μL, 100 μL, 200 μL, 300 μL, and 500 μL as the final volume of each probe was 500 µL. To 500 μL of the tested solution, 500 μL of the freshly prepared solution of 0.1 mM DPPH in methanol were added, the samples were incubated in the dark for 30 min, and then the extinction was measured at λ517 nm. A mixture of the solution of DPPH and methanol 1:1 was used as a control.

The percentage of DPPH scavenger activity was determined as follows

(1) $$Antioxidant\;activity\;\left(\%\right)=\frac{A_{control}-A_{sample}}{A_{control}}\times 100\;$$

Standard calibration curve was prepared using a stock solution of ascorbic acid in methanol (0.568 mM) for the preparation of different solutions, and the effect against DPPH radicals was expressed as mM ascorbic acid. Next, the maximal inhibition of DPPH radicals by ascorbic acid was assumed as effect 1, and activities of WWs were presented as part of this activity.

##### ABTS^+^ Assay

ABTS was oxidized using potassium persulfate to yield cation radical (ABTS^+^) at 743 nm. In the presence of hydrogen donating antioxidants, the solution was decolorized and the absorbance at 743 nm was decreased. A method, described by Re et al. (1999) [52] with a slight modification, was applied. ABTS^+^ was prepared by mixing 7 mM ABTS (in ddH_2_O) with 2.45 mM potassium persulfate (in ddH_2_O) and the mixture was allowed to stay in the dark at room temperature for 12–16 h before use. Prior to testing, the solution was diluted in methanol (2 mL ABTS^+^ + 58 mL methanol, where the absorbance should be 1.1 ± 0.02. To 75 μL of the sample, 1425 mL of diluted ABTS^+^ solution was added and the absorbance after 15 min of incubation at 37 °C against methanol was measured. A blank sample containing 75 μL ddH2O instead of the sample was measured also against methanol. The results were expressed as Trolox equivalents (mmol Trolox equivalents per mL solution of wastewater) using a calibration curve (obtained at different concentrations of Trolox: 0.1; 0.2; 0.3; 0.4; 0.5 mM, dissolved in methanol). Next, the maximal inhibition of ABTS^+^ by Trolox was assumed as effect 1, and activities of WWs were presented as part of this activity.

##### Inhibition of Superoxide (O^2−^) Generation

The NBT test was used for this purpose. Generation of ^·^O^2−^ was photochemically determined in medium containing: 50 mM potassium phosphate buffer, pH 7.8; 1.17 × 10⁶ M riboflavin; 0.2 mM methionine; 2x10⁵ M KCN; 5.6 × 10⁵ M nitro-blue tetrazolium (NBT). The generated ^·^O^2−^ reduced NBT to form blue-colored formazan, whose maximum absorption is at 560 nm. The absorption was measured in the presence of increasing wastewater concentrations [53].

The decrease in absorption indicated the O^2−^ scavenger effect of the tested substances, which was calculated as follows:

##### Inhibition of O^2−^ generation


(2) $$\%=\left(1-\frac{E_{s}}{E_{c}}\right)\times 100$$

*E_s_* is the absorption of media with the tested wastewater and *E_c_* is the absorption of the controls (without wastewater). After that, the maximal inhibition (95.9%) of 0.07 mM ascorbic acid was accepted as 1, and activities of WWs were presented as part of it.

### 4.3. Antibacterial Properties of Wastewater against Human Pathogens and Their Mode of Action

#### 4.3.1. Test Microorganisms

For evaluation of the antimicrobial activities of wastewater, the following test microorganisms were used: *Escherichia coli* ATCC 35218 (American Type Cell Culture Collection, USA), *Staphylococcus aureus* ATCC 29213, *Pseudomonas aeruginosa* ATCC 27853, and the fungus *Candida albicans* 562 from the SAIM-BAS collection.

#### 4.3.2. Culture Medium and Growth Conditions

The cultivation of *P. aeruginosa* and *C. albicans* was performed on Brain Heart Infusion Broth and Agar (BHIB, GM210, resp. BHIA, M1611, HiMedia, India). *S. aureus* and *E. coli* grew on Mueller Hinton Agar and Broth (MHA, M173, resp. MHB, M391, HiMedia, India). They were cultivated at 37 °C for 18 h. The microbiological methods were performed under sterile conditions into a Faster MSC Class II laminar box (SafeFAST Classic 212, Ferrara, Italy).

#### 4.3.3. Minimal Inhibitory (MIC) and Bactericidal (MBC) Concentrations

The antimicrobial activities of wastewaters were determined by the broth microdilution method according to ISO 20776-1:2006 and as described before [54,55]. Briefly, the bacterial inoculums with concentration 105 CFU/mL were added to 96-well plates containing MHB or BHIB loaded with twofold serial dilutions of wastewaters. Plates were incubated at 37 °C for 18 h. According to EUCAST (European Committee on Antimicrobial Susceptibility Testing) requirements, we used gentamicin for the test bacteria and amphotericin B for *C. albicans*. Experiments were performed in triplicate. MICs and MBCs were determined, as described before [54].

#### 4.3.4. Dehydrogenase (DEHA) Activity

The DEHA activity of the test microorganisms was performed by an MTT test [3-(4,5-dimethyl thiazolyl-2)-2,5- diphenyltetrazolium bromide, M2128-1 G, Sigma-Aldrich], as described before [54]. The method is based on the reduction of the MTT dye by the membrane located bacterial enzyme NADH:ubiquinone reductase (H+-translocation) to insoluble formazan crystals. Briefly, the treated and untreated bacterial/fungal cells were incubated for 2 h with MTT dye (final concentration 0.05 mg/mL). The formed formazan crystals were dissolved with 5% HCOOH in isopropanol. Absorption was measured on ELISA reader (BioTek Elx800, Instruments, Inc., Winooski, VT, USA) at 550 nm (reference 690 nm) against a blank solution.

### 4.4. Cytotoxicity of Wastewaters

#### 4.4.1. Cell Lines and Culture Conditions

As a model for “normal” non-cancer cells, transformed, immortalized, non-tumorigenic, and adherent cell lines CCL-1TM (mouse fibroblasts, NCTC clone 929) and HEK-293 (human embryonic kidney cells) were used. CCL-1 was purchased from the ATCC and HEK-293 was bought from the German collection of microorganisms and cell lines (DSMZ). CCL-1 cell line is recommended as a standard in Annex C of ISO 10993-5:2009 [30]. Cells were maintained in a controlled environment as previously described in [56] at 37 °C and a humidified atmosphere containing 5% CO_2_. The cell medium was Eagle’s MEM 2 with mM L-glutamine and Pen/strep for both cell lines but for CCL-1 cells it was supplemented with 10% heat-inactivated horse serum, while for HEK-293 10% heat-inactivated fetal bovine serum was added.

#### 4.4.2. In Vitro Cytotoxicity Evaluation (MTT Assay) on Cell Lines

Cytotoxicity of the wastewaters was assessed with the help of the MTT test, performed according to Annex C, ISO 10993-5:2009 [30]. The protocol follows that previously described in [56]. Briefly, 96-well flat-bottom microtiter plates were inoculated with the cell lines and after a 24 h incubation for entering the log phase, they were treated with the target wastewaters. Notably, there was an exposure time after treatment for 24 and 48 h as well as the incubation for 72 h. As a next step, MTT solution was added and after a 2 h incubation and solvation of the formed formazan crystals with acidified 2-propanol, absorbance was measured at 540 nm (reference filter at 690 nm) on a microplate reader ELx800 (BioTek Instruments, Inc., Winooski, VT, USA). The maximum tolerated concentrations (MTC) were calculated by GraphPad Prizm (version 6.01 for Windows, GraphPad Software Inc., San Diego, CA, USA) and the model log (inhibitor) vs. normalized response—variable slope (Y = 100/(1 + 10^((LogIC_50_-X) *HillSlope) was used [56]. The half maximal inhibitory concentration (IC_50_) values after 72 h of treatment were calculated using the MAPLE^®^ software of symbolic mathematics and expressed as µg/mL polyphenols. The latter were determined based on the calculated IC_50_ as percent wastewater (*v/v*) and the contents of polyphenols (mg/mL) of each wastewater.

### 4.5. Antiviral Properties of Wastewaters against the Replication of Herpes Simplex Virus Type-1 (HSV-1)

#### 4.5.1. Cells

MDBK cells were obtained from National Bank for Industrial Microorganisms and Cell Cultures, Sofia. The cell lines were grown in DMEM medium containing 10% fetal bovine serum (FBS-HI-11A, Capricorn Scientific, Ebsdorfergrund, Germany), supplemented with 10 mM HEPES buffer (HEP-B, Capricorn Scientific, Ebsdorfergrund, Germany) and antibiotics (penicillin 100 IU/mL, streptomycin 100 μg/mL) in a CO_2_ incubator (HERA cell 150, Heraeus, Germany) at 37 °C/5% CO_2_.

#### 4.5.2. Viruses

##### HSV-1 Victoria Strain

The HSV-1 Victoria strain was received from Prof. S. Dundarov, National Center of Infectious and Parasitic Diseases, Sofia. The virus was replicated in monolayer MDBK cells in a maintenance solution DMEM Gibco BRL, Paisley, Scotland, UK, plus 0.5% fetal bovine serum Gibco BRL, Scotland, UK. The infectious titer of the stock virus was 10^8.0^ CCID_50_/mL.

##### HSV-1 Resistant to ACV

HSV-1 resistant to ACV, strain R-100 (Prof. G. Palu, Institute of Microbiology, University of Padua, Italy, given to us by the Laboratory of Virology, Sofia University „St. Kliment Ohridski”) is characterized by a mutation in the gene encoding the enzyme TK, which has altered substrate specificity (TK a, HSV-1). The infectious titer of the stock virus was 10^6.0^ CCID50/mL.

#### 4.5.3. Cytotoxicity Assay

Confluent monolayer cell cultures (MDBK) in a 96-well plate were treated with a culture medium containing a falling concentration of the wastewaters. The 50% cytotoxic concentration or half maximal cytotoxic concentration (CC_50_) was defined as the concentration that reduced the cell viability by 50% for 48 h when compared to the untreated control.

The maximum tolerable concentration (MTC) of the wastewaters was also determined as that concentration which does not affect the cell monolayer and at which the sample looks like the cells in the untreated control.

#### 4.5.4. Antiviral Activity Assay

Cytopathic effect (CPE) inhibition test was used to assess the antiviral activity of the wastewaters. Confluent cell monolayer in 96-well plates was infected with 100 cell culture infectious dose 50% (CCID_50_) in 0.1 mL (Victoria or R-100 strain). After 60 min of virus adsorption, wastewaters were added in various concentrations, and cells were incubated for 48 h at 37 °C. The cytopathic effect was determined using a neutral red uptake assay and the percentage of CPE inhibition for each concentration of the test sample was calculated using the following formula

(3) $$\%CPE=\frac{{\mathrm{OD}}_{\mathrm{test}\;\mathrm{sample}}-{\;\mathrm{OD}}_{\mathrm{virus}\;\mathrm{control}}}{{\mathrm{OD}}_{\mathrm{toxicity}\;\mathrm{control}}-{\mathrm{OD}}_{\mathrm{virus}\;\mathrm{control}}}\times 100$$

where OD_test sample_ is the mean value of the ODs of the wells inoculated with virus and treated with the test sample in the respective concentration, OD_virus control_ is the mean value of the ODs of the virus control wells (with no wastewater in the medium), and OD_toxicity control_ is the mean value of the ODs of the wells not inoculated with the virus but treated with the corresponding concentration of the wastewaters. The 50% inhibitory concentration (IC_50_) was defined as the concentration of the material that inhibited 50% of viral replication when compared to the virus control. The selectivity index (SI) was calculated from the ratio CC_50_/IC_50_.

### 4.6. Mathematical Modeling of the In Vitro Cytotoxicity Data

For calculation of the half maximal (50%) inhibitory concentration (IC_50_) values from the MTT assay after 72 h of incubation of the cell lines CCL-1 and HEK-293 with wastewaters, a non-linear regression procedure coded in MAPLE^®^ software of symbolic mathematics was applied. This procedure is based on weighted least squares statistical criterion as an objective function of the search and was developed by our group as published before [57]

F_a_/F_u_=(Dose/D_m_)^m^ (4)

where F_a_ stands for the affected fraction; F_u_ stands for the unaffected fraction (1 − Fa) =F_u_; Dose stands for the concentration of the wastewater sample; D_m_ stands for the concentration achieving a median-effect (D_m_=IC_50_ in our case); m is the slope of the median-effect plot (shape of the dose-effect curve).

### 4.7. Statistical Analysis

The cytotoxicity experiments were performed in triplicate whereby three independent assays were used (*n* = 3). Data from the in vitro cytotoxicity assay were collected from at least four repetitions for every concentration within each assay. The statistical evaluation of the in vitro cytotoxicity data was performed using One-way ANOVA (GraphPad Prizm, version 6.01 for Windows, GraphPad Software Inc., San Diego, CA, USA). Each of the tests for antiviral properties was performed in triplicate to quadruplicate, with four cell culture wells per test sample. The SI values of the wastewaters were analyzed statistically by using the two independent sample Student’s t-test. The CC_50_ and IC_50_ values were presented as mean ± SD. A *p*-value < 0.05 was considered statistically significant.

## Figures and Tables

**Table 1:** Median effective concentration of antiradical effects of wastewaters obtained after the distillation of rose oil of four Bulgarian oil-bearing roses—*R. damascena* Mill., *R. alba* L., *R. centifolia* L., and *R. gallica* L.

	Roses WW	*WW* from *R. centifolia* L.	WW from *R. gallica* L.	WW from*R. damascena* Mill.	WW from *R. alba* L.
Antiradical Activity					
DPPH scavenging					
HillSlope		0.61	1.57	1.74	2.52
EC_50_		0.14 *	0.07 *	0.09 *	0.07 *
R (correlation coefficient)		0.97	0.99	0.95	0.95
ABST^+^ scavenging					
HillSlope		0.139	1.67	1.44	1.56
EC_50_		0.84 *	0.86 *	0.92 *	0.69 *
R (correlation coefficient)		0.99	0.99	0.99	0.99
^•^O_2_^−^ scavenging					
HillSlope		1.78	1.59	3.20	2.55
EC_50_		0.04 *	0.04 *	0.04	0.04 *
R (correlation coefficient)		0.99	0.99	0.99	0.99

Legend: EC_50_ 50%—half maximal effective concentration; R—coefficient of correlation; * volumetric concentration (% *v/v*).

**Table 2:** Minimal inhibitory concentration, minimal bactericidal concentration (MIC/MBC), and DEHA activity expressed as mg QuE/mL.

	Rose WWs	*WW fromR. alba* L.	*WW fromR. damascena* Mill.	*WW fromR. centifolia* L.	*WW fromR. gallica* L.	Controls (Reference Drugs)
Microorganisms						
*S. aureus*						Gentamicin
MIC		3.8	3.6	1.9	4.36	0.25
DEHA (%±SD)		32.53 ± 0.03	29.4 ± 0.01	45.28 ± 0.05	35.8 ± 0.03	
MBC		>3.8	>3.6	>3.9	>4.47	
*P. aeruginosa*						Gentamicin
MIC		>3.8	>3.6	>3.9	>4.36	0.5
DEHA (%±SD)		*-*	*-*	-	*-*	
MBC		>3.8	>3.6	>3.9	>4.36	
*E. coli*						Gentamicin
MIC		>3.8	>3.6	>3.9	>4.36	2.0
DEHA (%±SD)		*-*	*-*	-	*-*	
MBC		>3.8	>3.6	>3.9	>4.36	
*C. albicans*						Amphotericin B
MIC		>3.8	>3.6	>3.9	>4.36	1.25
DEHA (%±SD)		*-*	-	-	-	
MBC		>3.8	>3.6	>3.9	>4.36	

**Table 3:** Median inhibitory concentrations and maximum tolerated concentrations of wastewaters obtained from different rose species in non-tumorigenic cell lines after 24 and 48 h of incubation.

Wastewaters From:	Model Parameters	Incubation Time [h]	CCL-1	HEK-293
*R. damascena* Mill.	IC_50_	24	6.5% * (= 467 µg QuE/mL) **	2.6% (= 183 µg QuE/mL)
		48	1.4% (= 102 µg QuE/mL)	1.9% (= 137 µg QuE/mL)
	MTC	24	2.2% (= 158 µg QuE/mL)	2.2% (= 158 µg QuE/mL)
		48	0.4% (= 29 µg QuE/mL)	1.5% (= 108 µg QuE/mL)
*R. alba* L.	IC_50_	24	3.4% (= 255 µg QuE/mL)	2.2% (= 168 µg QuE/mL)
		48	0.8% (= 63 µg QuE/mL)	1.2% (= 93 µg QuE/mL)
	MTC	24	1.7% (= 129 µg QuE/mL)	1.7% (= 129 µg QuE/mL)
		48	0.2% (= 15 µg QuE/mL)	0.9% (= 68 µg QuE/mL)
*R. centifolia* L.	IC_50_	24	11.1% (= 867 µg QuE/mL)	2.3% (= 176 µg QuE/mL)
		48	1.0% (= 80 µg QuE/mL)	1.9% (= 146 µg QuE/mL)
	MTC	24	3.4% (= 265 µg QuE/mL)	1.8% (= 140 µg QuE/mL)
		48	0.4% (= 31 µg QuE/mL)	1.6% (= 125 µg QuE/mL)
*R. gallica* L.	IC_50_	24	5.3% (= 459 µg QuE/mL)	2.4% (=208 µg QuE/mL)
		48	1.0% (=86 µg QuE/mL)	1.3% (= 114 µg QuE/mL)
	MTC	24	1.7% (= 148 µg QuE/mL)	2.0% (= 174 µg QuE/mL)
		48	0.4% (= 35 µg QuE/mL)	1.0% (= 87 µg QuE/mL)

Legend: *—volumetric concentration in (%); **—(µg QuE/mL) = concentration of total polyphenols determined as quercetin equivalents.

**Table 4:** Cytotoxicity (on MDBK cells) and antiviral activity of the wastewater from rose oil production against HSV-Victoria strain and acyclovir resistant R-100 strain after 48 h of incubation.

Roses WW	Cytotoxicity		Antiviral Activity			
	CC_50_ (%)	MTC (%)	Victoria Strain		R-100 Strain	
			IC50 (%)	SI	IC_50_ (%)	SI
*R. alba* L.	2.5 ± 0.2	0.1	0.43 ± 0.02	5.8	0.55 ± 0.05	4.5
*R. damascene* Mill.	2.3 ± 0.1	0.1	0.52 ± 0.02	4.4	0.63 ± 0.04	3.6
*R. centifolia* L.	1.4 ± 0.09	0.1	0.15 ± 0.01	9.3	0.16 ± 0.01	8.7
*R. gallica* L.	1.5 ± 0.08	0.1	0.12 ± 0.01	12.5	0.18 ± 0.02	8.3

Legend: CC_50_—half maximal cytotoxic concentration; MTC—maximum tolerable concentration; IC_50_—half-maximal inhibitory concentration; SI—selectivity index.

## Data Availability

The data in this study and the samples of wastewaters are available from the authors.
